# Deltamethrin Poisoning Mimicking Organophosphate Poisoning: A Case Report

**DOI:** 10.7759/cureus.34303

**Published:** 2023-01-28

**Authors:** Waseem M Ilyas, Gajanan Chavan, Charuta Gadkari

**Affiliations:** 1 Emergency Medicine, Jawaharlal Nehru Medical College, Datta Meghe Institute of Higher Education and Research, Wardha, IND

**Keywords:** toxicology and envenomation, insecticide poisoning, unknown poisoning, smell of organophosphate, fasciculations, atropine challenge test, organophosphate poisoning, organophosphate, deltamethrin poisoning, deltamethrin

## Abstract

Deltamethrin is a newer class of insecticide used on crops, pets, and livestock, in home pest control, and malaria vector control belonging to the synthetic pyrethroid group, which is being promoted in the place of organophosphate compounds due to the harmful and persistent effects of the latter. Unfortunately, as its usage increased, so has the number of poisoning cases associated with deltamethrin. Fortunately, the mortality in deltamethrin poisoning cases is low. However, deltamethrin poisoning causes signs and symptoms similar to the clinical features of organophosphate poisoning. This case report is of a 20-year-old man who consumed an unknown substance in a suicidal attempt and presented with clinical signs of organophosphate toxicity. Later the compound was identified as deltamethrin. This case report adds to the medical literature on deltamethrin poisoning. It showed that apart from the similarity in their clinical features in toxicity, deltamethrin can even give a positive result on atropine challenge tests like organophosphate and that the fasciculations induced by deltamethrin may be temporary. This case report will also benefit the clinician in unknown compound poisoning cases as it shows that the clinician can suspect deltamethrin toxicity alongside organophosphate toxicity in the differential diagnosis when the atropine challenge test gives a positive result.

## Introduction

Deltamethrin is a pesticide belonging to the synthetic pyrethroid group. It was introduced as a replacement for organophosphate and organochlorinated pesticides, which were harmful and persistent. It is used as an insecticide on crops, a pesticide for pets and livestock, in home pest control, and malaria vector control [1]. Poisoning with deltamethrin is most commonly seen in agricultural workers, deltamethrin packaging workers, and following deliberate suicidal attempt ingestions. Some of its clinical features include fasciculations, muscle cramps, twitches, convulsions, pulmonary edema, bronchospasm, altered sensorium, diarrhea, rhinorrhoea, lacrimation, salivation, myosis, tachycardia, nausea, vomiting, headache, dizziness are also seen in organophosphate poisoning and can lead to pyrethroid poisoning being misinterpreted as organophosphate poisoning especially if the compound is unknown. Clinicians should be aware of deltamethrin poisoning as its features mimic organophosphate poisoning, but the treatment differs. There is no specific antidote for deltamethrin poisoning. The treatment of pyrethroid poisoning is symptomatic with supportive care. Most of the symptoms and signs resolve in five to six days. In patients with clinical features of salivation and pulmonary edema, atropine can be given in small doses to decrease secretions. Although very few cases of death following deltamethrin poisoning have been reported, the prognosis is good even in severe poisoning cases [2].

The atropine challenge test is an atropine requirement indicator test used in organophosphate poisoning, and patients with a positive test have a significantly low level of serum cholinesterase and have a response to atropine. If the test result is negative, the patient may not need atropine. In organophosphate poisoning, atropine is not indicated if the patient has no clinical signs. If the patient presents with atypical symptoms or the physician has doubts about the diagnosis, an atropine challenge test is done [3].

We describe a case where the patient had ingested an unknown chemical to commit suicide and had clinical features similar to organophosphate poisoning. Our patient’s case was a dilemma in diagnosis and treatment as the compound was unknown. There were clinical features resembling organophosphate poisoning; however, the compound was later identified as deltamethrin.

As deltamethrin is being promoted as a safer alternative to organophosphate compounds, the poisoning cases of deltamethrin are also increasing [4]. So it is essential to add as much new information to the literature. This case report will also benefit the clinician in unknown compound poisoning cases as it shows that the clinician can suspect deltamethrin and organophosphate as toxic compounds when it gives a positive atropine challenge test.

## Case presentation

A 22-year-old male was brought to the Emergency Department (ED) by relatives with a history of consuming about 100 ml of a chemical about one and a half hours back in an attempt to commit suicide. The chemical was not brought to the ED and relatives did not know the chemical’s brand name or content. He has had about 10 episodes of vomiting since then.

On physical examination, vitals were as follows: pulse rate: 102 per minute, blood pressure: 120/80 mmHg, respiratory rate: 16 per minute, temperature: 97.5 °F, and oxygen saturation: 100% on room air. On auscultation, his lungs were clear and had equal air entry bilaterally. His heart rate was regular with normal S1 and S2, and there were no appreciable murmurs, rubs, or gallops. The abdomen was soft, non-tender, and non-distended, with normal bowel sounds. Neurological examination was normal. Pupils were normal in size bilaterally and reactive to light. There were fasciculations of his calf muscles and a smell resembling the smell of organophosphate compounds. He had no previous history of suicidal attempts or psychiatric illnesses.

As organophosphate poisoning was suspected due to the presence of fasciculations and smell resembling organophosphate compounds, an atropine challenge test was done with 1 mg of atropine. The test was positive, with the heart rate rising from 105 per minute to 152 per minute. The positive atropine challenge test further increased the suspicion of organophosphate poisoning. It was thus assumed to be organophosphate poisoning, and the patient was started on a minimal dose of 2 mg atropine and starting dose of 30 mg per kg pralidoxime in 100 ml normal saline over 20 minutes as per routine treatment followed for organophosphate compound poisoning [5]. Gastric lavage was also done. After some time, relatives brought the compound, which was identified as deltamethrin, and the particular brand was advertised as an anti-tick animal medication. He had consumed around 100 ml of deltamethrin 12.5 mg per ml. It was decided to stop pralidoxime, which had already been almost wholly infused intravenously, and start supportive treatment. The fasciculations lasted only for about 30 minutes since his arrival. He was admitted to the intensive care unit and was conservatively treated with pantoprazole and intravenous fluids. His complete blood counts, kidney and liver function tests, coagulation profile, and electrocardiogram (ECG) were within normal limits. Serum cholinesterase levels were also normal. There were no complications during his four-day stay in the hospital. A psychiatrist evaluation was also done, and he was diagnosed with depression disorder following his marriage request being denied by his girlfriend. He was discharged on the fourth day with escitalopram and clonazepam prescribed by the psychiatrist.

## Discussion

Deltamethrin is a pesticide belonging to the synthetic pyrethroid group. It was introduced as a replacement for organophosphate and organochlorinated pesticides due to the harmful and persistent features of the latter. It is used as an insecticide on crops, a pesticide for pets and livestock, home pest control, and malaria vector control. Deltamethrin is chemically stable, non-volatile, lipophilic, and readily soluble in organic solvents but insoluble in water. Thus its formulations are suspensions rather than solutions. Due to it being a non-volatile and lipophilic compound, the risk of inhalation poisoning is low and is readily absorbed orally and through the skin. Deltamethrin plasma concentration is maximum at about two hours after exposure. It gets hydrolyzed in our body and is excreted mainly in the urine and partly via feces [1]. The other popularly used pyrethroids are fenvalerate and cypermethrin. These synthetic pyrethroids have low toxicity in mammals and leave no residue in nature [2].

**Figure 1:**
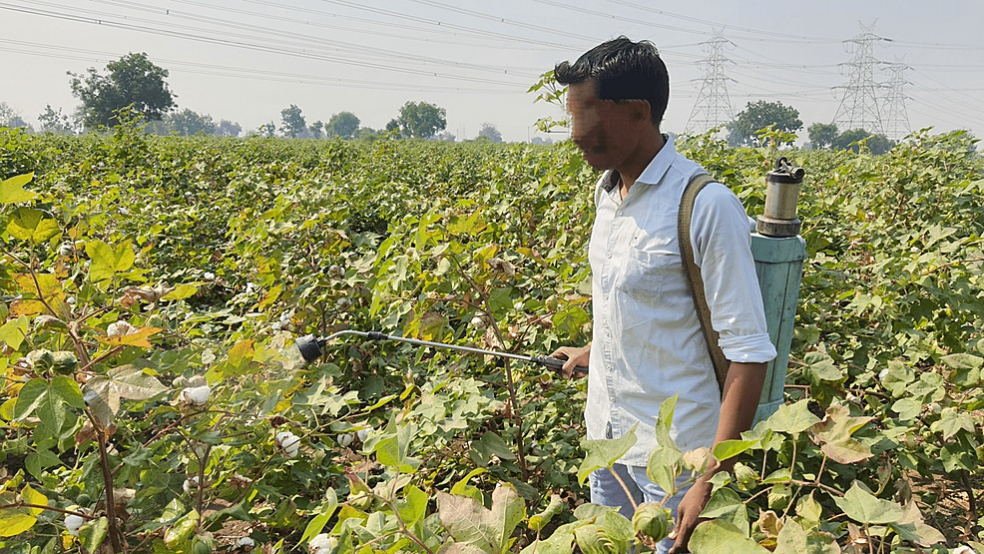
A farmer spraying deltamethrin on his crops

Deltamethrin poisoning occurs mainly as occupational exposure in agricultural workers (Figure 1), deltamethrin packaging workers, and following deliberate suicidal attempt ingestion. Occupational exposure occurred due to inadequate protective measures like lack of personal protection, clearing blocked spray machines by mouths and hands, improper application like exposure for a long duration, using high concentrations, spraying against wind and accidental ingestion. Local exposure to skin, eyes and upper airways can cause erythema, burning sensations, development of red miliary papules, rhinorrhoea, lacrimation, sneezing, and pruritus, especially of the face and perioral area, which may last for two days followed by desquamation of the exposed area of skin, photophobia, conjunctival congestion, bronchospasm [1,2]. There may also be headache, dizziness, nausea, fatigue, anorexia, diarrhea, paresthesia of lower limbs, mouth, tongue, and transient electroencephalogram (EEG) changes. Tingling sensations may last up to two days. Muscle fasciculations and convulsions can also occur in severe cases. Ingestion of deltamethrin can cause nausea, vomiting, headache, dizziness, fatigue, photophobia, salivation, myosis, tachycardia, paresthesia, muscle cramps, twitches, fasciculations and even loss of consciousness [1,2]. Large ingestions of more than 500 mg can cause severe toxicity with convulsions with a frequency of even up to 30 times a day and pulmonary edema [2]. The solvent of deltamethrin, xylene, can also cause hepatotoxicity and gastrointestinal disturbances. Skin irritation can also occur in people using deltamethrin-treated bednets. Skin manifestations are early signs of local exposure and can be easily treated by stopping the exposure. Mortality has also been reported in severe poisoning cases with convulsions. Long-term effects of poisoning and carcinogenicity have not been observed [1].

Our patient’s case was a dilemma in diagnosis and treatment as the compound was unknown. The smell resembling organophosphate compounds and the presence of fasciculations favor the compound being an organophosphate. The positive atropine challenge test further strengthened this suspicion. Thus, it was decided to treat it as organophosphate poisoning. Atropine was given at a minimal dose of 2 mg, and pralidoxime was given at starting dose of 30 mg per kg as fasciculations in organophosphate poisoning are relieved by oximes by restoring acetylcholinesterases at nicotinic sites [5].

The smell of pyrethroids is similar to organophosphate compounds because of the similar hydrocarbon solvents used for both these compounds. This feature, along with similar symptoms in acute poisoning such as fasciculations, muscle cramps, twitches, convulsions, pulmonary edema, bronchospasm, altered sensorium, diarrhea, rhinorrhoea, lacrimation, salivation, myosis, tachycardia, nausea, vomiting, headache, dizziness can lead to pyrethroid poisoning being misinterpreted as organophosphate poisoning especially if the compound is unknown. There is no inhibition of cholinesterase by pyrethroids [2,6]. Clinicians should be aware of deltamethrin poisoning as its features mimic organophosphate poisoning, but the treatment differs.

There is no specific antidote for deltamethrin poisoning. The treatment of pyrethroid poisoning is symptomatic with supportive care. Most of the symptoms and signs resolve in about six days. Gastric lavage should be given as soon as possible. In cases with salivation, pulmonary edema atropine can be given to decrease secretions. The atropine dose needed is mostly less than 10 mg. Although very few cases of death following deltamethrin poisoning have been reported, the prognosis is good even in severe poisoning cases [2]. 

In organophosphorus poisoning, atropine is not indicated if the patient has no clinical signs. If the patient presents with atypical symptoms or the physician doubts the diagnosis, an atropine challenge test is done. The atropine challenge test is an atropine requirement indicator test, and patients with a positive test have a significantly low level of serum cholinesterase and have a response to atropine. If the test result is negative, the patient may not need atropine. In the atropine challenge test, the baseline heart rate is first recorded, then 1mg of atropine is given intravenously, and its effect on heart rate is tracked. Atropine usually causes an increase in heart rate; however, in organophosphate poisoning patients who require atropine, this increase is more than 20% of the baseline or more than 30 beats per minute [3]. In our patient, the atropine challenge test was positive even though the compound was deltamethrin. At the time of presentation to the ED, as the compound was unknown, this positive result further increased our suspicion of organophosphate poisoning. The importance of this case report is that it shows deltamethrin can cause a positive atropine challenge test similar to organophosphate compounds; this has not been reported before in medical literature. Also, the patient had fasciculations and a smell resembling organophosphate, per previous deltamethrin studies. The fasciculations in deltamethrin toxicity could be short-lived, as was in our case. The patient also had a good prognosis even after massive ingestion of deltamethrin which could have been because of the multiple episodes of vomiting he had, which could have decreased the quantity available for absorption or because the prognosis is good even in severe cases of deltamethrin poisoning [2].

## Conclusions

Deltamethrin is a known mimic of organophosphate with regard to the symptoms of their toxicity. The clinical features of deltamethrin poisoning that are similar to organophosphate poisoning are fasciculations, muscle cramps, twitches, convulsions, pulmonary edema, bronchospasm, altered sensorium, diarrhea, rhinorrhoea, lacrimation, salivation, myosis, tachycardia, nausea, vomiting, headache, dizziness. The significance of this case report is that it showed that deltamethrin could cause a positive atropine challenge test similar to organophosphate compounds, and this finding has not been reported before in medical literature. Also, our findings that the patient had a smell resembling organophosphate compounds and the presence of fasciculations are in accordance with previous studies on deltamethrin toxicity. The fasciculations in deltamethrin toxicity could be short-lived, as in our patient. As deltamethrin is being promoted as a safer alternative to organophosphate compounds, the poisoning cases of deltamethrin are also increasing. So it is essential to add as much new information emerging to the literature. This case report will also benefit the clinician in unknown compound poisoning cases, as the clinician can now suspect deltamethrin along with organophosphate as the toxic compound when the atropine challenge test gives a positive result.
